# Evaluating the Propagation of Uncertainties in Biologically Based Treatment Planning Parameters

**DOI:** 10.3389/fonc.2020.01058

**Published:** 2020-07-21

**Authors:** Miriam A. Barry, Mohammad Hussein, Giuseppe Schettino

**Affiliations:** ^1^National Physical Laboratory, Metrology for Medical Physics Department, Teddington, United Kingdom; ^2^Department of Physics, University of Surrey, Guildford, United Kingdom

**Keywords:** normal tissue complication probability (NTCP), tumor control probability (TCP), uncertainty, biologically based treatment planning, biological optimization

## Abstract

Biologically based treatment planning is a broad term used to cover any instance in radiotherapy treatment planning where some form of biological input has been used. This is wide ranging, and the simpler forms (e.g., fractionation modification/optimization) have been in use for many years. However, there is a reluctance to use more sophisticated methods that incorporate biological models either for plan evaluation purposes or for driving plan optimizations. This is due to limited data available regarding the uncertainties in these model parameters and what impact these have clinically. This work aims to address some of these issues and to explore the role that uncertainties in individual model parameters have on the overall tumor control probability (TCP)/normal tissue complication probability (NTCP) calculated, those parameters that have the largest influence and situations where extra care must be taken. In order to achieve this, a software tool was developed, which can import individual clinical DVH's for analysis using a range of different TCP/NTCP models. On inputting individual model parameters, an uncertainty can be applied. Using a normally distributed random number generator, distributions of parameters can be generated, from which TCP/NTCP values can be calculated for each parameter set for the DVH in question. These represent the spread in TCP/NTCP parameters that would be observed for a simulated population of patients all being treated with that particular dose distribution. A selection of clinical DVHs was assessed using published parameters and their associated uncertainties. A range of studies was carried out to determine the impact of individual parameter uncertainties including reduction of uncertainties and assessment of what impact fractionation and dose have on these probabilities.

## Introduction

Radiobiology has played a critical role in clinical radiotherapy for many years, and it is common practice to use radiobiological methods, for example, to account for different fractionation regimes and modalities in combined treatment (such as combined external beam radiotherapy and brachytherapy in gynecological treatments) (1) and to account for interruptions in treatment (2). Following the significant technological development of the last decade, which has resulted in a variety of methods for the delivery of precise radiation doses, there is now a drive to implement radiobiological methods in either the evaluation of treatment plans (for plan comparison for an individual patient) or for triaging patients who would benefit from more advanced radiotherapy techniques, e.g., protons (3) or the actual optimization of plans. Moreover, the use of radiobiological models for treatment plan optimization is an important first step for the development of truly personalized radiotherapy. This would allow full exploitation of the therapeutic power of radiation and the advances in genomic testing while safeguarding the more radiosensitive individuals.

The Task Group document from the AAPM, report 166 (4, 5) provides an outline describing biologically based treatment planning including descriptions of commonly used models and how different treatment planning systems (TPS) implement these. They also provide guidelines for implementation and quality assurance (QA) of such systems and vision for the future. A coherent explanation of the different levels of biological optimization was described by Nahum et al. (6). They outlined the different levels, which ranged from very simple methods (level 1) included in trials, such as IDEAL-CRT and I-Start, which individualize/escalate prescription levels based on organ at risk dose, to level 5, where they envisage such techniques being employed that would take into account a patient's individual (*not* based on population data) biology. The different levels coined by Nahum et al. are paraphrased from the original publication (6) below. The reader is directed to their publication for further details on this subject.

**Table:** 

Level 0:	no biological optimization.
Level I:	individualization of prescription dose for specified level of toxicity [i.e., dose escalated in plans where possible using fixed normal tissue control probability (NTCP) level for the organ at risk (OAR)].
Level II:	the same as above, but the number of fractions is adjusted as well as the prescription dose based on an isotoxic basis.
Level III:	biological cost functions used in the actual optimization of the dose distribution. Equivalent uniform dose (EUD), TCP, and NTCP parameters are used alongside conventional DVH parameters used in the optimization (e.g., Dmax, D99, V50%, mean).
Level IV:	individual patient-specific data is used in the optimization of the patient's plan (e.g., use of functional imaging to highlight areas of hypoxia and other areas of increased radio-resistance).
Level V:	using individual patient biology to optimize dose prescriptions in conjunction with any of the other levels.

We are currently at, or are approaching, level III where TPS are now incorporating biological models for either evaluation or optimization purposes. One of the benefits in using TCP or NTCP models is that a single value can be used in place of an array of dosimetric parameters describing points along a DVH curve. However, it is critical to assess the uncertainty affecting such values and what the key elements underpinning such uncertainty are. Different companies and TPS systems employ different formalisms and algorithms despite adopting the same radiobiological models, resulting in fundamental differences for the final calculations of the TCP/NTCP values and related uncertainties. An accurate understanding of how the uncertainties associated with the input parameters impact the final NTCP or TCP values is paramount and will support the increasing use of such approaches in planning radiotherapy treatments. Moreover, understanding how uncertainties are propagated in the TCP and NTCP calculations will identify the input parameters, which will need to be better defined along with their acceptable level of uncertainty to guide pre-clinical research efforts.

The aim of this study was to assess the impact of uncertainties in individual parameters used in TCP/NTCP calculations. This was achieved using in-house software developed in MATLAB™ vR2017a (The MathWorks Inc., MA, USA) that generates a simulated population of parameters within the constraints of the input parameters and their uncertainties, from which TCP or NTCP values and their uncertainties can be calculated. Dose is supplied using the 1D dose distribution as described by the planning DVH for the structures in question. The simulated data is generated using MatLab™'s normally distributed random number generator. A set of values are generated that are normally distributed with a mean, standard deviation, and size as specified by the user. The impact from single-parameter and multiple-parameter uncertainties was assessed for a range of clinically acceptable prostate plans, focusing on both survival probability and the probability of rectal complications. A similar approach was taken by Zhang et al. (7) for epithelial pleural mesothelioma where they applied an uncertainty to either the alpha term for the TCP model used (8) or the D_50_ parameter of the NTCP model used, the Lyman Kutcher Burman (LKB) model (9–12). This was to simulate heterogeneity in radiosensitivity of a population over a set dose range and focusing on therapeutic ratio for the prescription dose set. Our approach used uncertainties in all model input parameters and was focused not on a prescription dose but the planned organ doses (physical and biological) from clinical plans. No uncertainty was applied to the dose; however, there was a natural variation in doses as a result of the uncertainties applied to those parameters used in the biological dose calculation. The resulting dose (for the rectum especially) was very specific to the individual anatomy and the resulting plan generated. Plans were selected to represent the full range of possible doses that might be encountered.

The data reported show how the proposed approach can quickly generate uncertainty levels for TCP and NTCP models (for individual dose distributions as calculated by a treatment planning system for clinically acceptable plans) taking into consideration the uncertainties in the input parameters. The approach was tested for the specific case of prostate treatment and using the Lind (13) and LKB (9–12) models for TCP and NTCP, respectively, and highlighted the key role that the D_50_ parameter plays in the overall uncertainties. The study also indicates a strong synergy between the input parameters with small uncertainties on a single parameter having an overall large effect on the variation in the TCP/NTCP values generated when combined with uncertainties from the other parameters. The approach can be considered a first step in the robustness validation of radiotherapy treatment planning based on biological optimization.

## Methods

### Software

Software was developed in MATLAB, which allowed the import of dose volume histograms from clinical plans from different TPS. Different input formats available were for DVHs from BioSuite (13), Eclipse™, Pinnacle™, and Raystation™. Dose and volume data were processed to allow the visualization of both cumulative and probability density histograms of the data. Using the linear quadratic formula, dose was converted into the equivalent dose if the treatment was given in 2 Gy fractions (LQED2) (14, 15) using the alpha/beta ratio (α/β) and the number of fractions as supplied by the user (see Equation 1). The α/β ratio is from the linear-quadratic relationship between cell survival and irradiated dose, *D*_*i*_ is the total dose per DVH dose-bin, and n is the number of fractions.

(1) $$\begin{array}{l} {LQED2}_{i}=\;D_{i}\cdot \frac{1+\frac{D_{i}/n}{\alpha /\beta }}{1+\frac{2}{\alpha /\beta }} \end{array}$$

It is possible, in the software, to incorporate an uncertainty on the α/β value entered. This is used to generate a normally distributed virtual distribution of alpha and beta parameters. This is done using the normally distributed random number generator in MatLab™, which generates a distribution of values of size n. The values are generated such that they have a mean and standard deviation that matches that supplied by the user. Using these parameters, simulated variations in the LQED2 are calculated. After calculation of the LQED2 variations for a DVH, the required radiobiological models can be selected for the TCP and NTCP analysis.

The TCP model used for this study was Lind's model (16). Equation (2) shows the model formula as displayed in AAPM report 166 (4) for use with doses converted into LQED2 using Equation (1). The γ parameter is the slope parameter, D_50_ is the dose at which there is a 50% probability of tumor control occurring, and D_i_ and v_i_ are dose-bin values and corresponding fractional volume obtained from the DVH, respectively.

(2) $$\begin{array}{l} P\left(D_{i}\right)=exp\left(-exp\left(e\gamma -\frac{D_{i}}{D_{50}}\left(e\gamma -ln\left(ln2\right)\right)\right)\right)\\ \;TCP=\;\overset{M}{\underset{i\;=\;1}{\prod }}P{\left(D_{i}\right)}^{v_{i}} \end{array}$$

The NTCP model used in this study was the LKB model (9–12) (see Equations 3–5), where effective dose (D_eff_) is the uniform dose, which gives the equivalent biological effect to the structure in question as the planned inhomogeneous dose distribution from the DVH. The volume parameter n describes how serial or parallel an organ is. D_i_ and v_i_ are dose-bin values and corresponding fractional volume obtained from the DVH. The m parameter describes the slope of the NTCP vs. dose relationship, and D_50_ is the dose at which 50% chance of complications occur. Similar to what was described for the α/β uncertainty, uncertainties in all the above user input parameters (i.e., D_50_, m, n) can be provided and are propagated to the final NTCP value calculated. TCP/NTCP values are collected for each simulation and the standard deviation calculated as a measure of the propagated uncertainty.

(3) $$\begin{array}{l} NTCP=\;\frac{1}{\sqrt{2\pi }}\int_{-\infty }^{t}e^{\frac{x^{2}}{2}}dx \end{array}$$

(4) $$\begin{array}{l} t=\frac{D_{eff}-D_{50}}{mD_{50}} \end{array}$$

(5) $$\begin{array}{l} D_{eff}={\left(\underset{i}{\sum }v_{i}D_{i}^{1/n}\right)}^{n} \end{array}$$

### Input Parameters

DVHs from a selection of clinically acceptable prostate plans were used for this study. The conventional vs. hypofractionated high-dose intensity-modulated radiotherapy for prostate cancer (CHHIP) trial (17) planning constraints were used, and the prescription was either 74 or 78 Gy to allow a range of doses (and, therefore, positions on the TCP curve) to be evaluated. The input parameters investigated were taken from the literature. Initial study parameters for rectal toxicity were from Lyman et al. (10): D_50_ = 7,500 cGy, m = 0.1, n = 0.1, and α/β = 300 cGy. A study from Marzi et al. (18) was used for the later analysis as this study provided uncertainty values with its published parameters. Parameters used were for the prediction of greater than, or equal to, G2 late toxicity of the rectum; D_50_ = 7,600 ± 190 cGy, α/β = 230±60 cGy, n = 0.12, and m = 0.15 (no uncertainties were provided for the n and m parameters). The TCP values used for the prostate PTV analysis were from Okunieff et al. (19) for T2 multi-institute macroscopic disease; the slope parameter γ was used for the slope parameter γ in the Lind formulism, γ = 1.16 and D_50_ = 4,518 cGy. Data from the CHHIP trial (17) was used for the prostate α/β parameter: α/β = 180 cGy.

### Analysis 1

For the first analysis, the uncertainty in each parameter was progressively increased to determine its specific impact on the final probability calculated. The analysis was performed by varying the uncertainty on one parameter at a time while assuming no error on the other parameters. This was carried out for both NTCP and TCP models investigating the rectum and for the prostate PTV. Analysis was performed using Lyman and Okunieff parameters for the NTCP and TCP calculation, respectively. For each model, in turn, and for each associated parameter, in turn, the MatLab code allowed simulated sets of parameters to be generated. These sets of input parameters were simulated such that the mean and standard deviation were as specified by the user. Using each value, in turn, from the simulated parameter set, an NTCP or TCP value was then calculated. Finally, a mean and standard deviation was calculated over the probabilities (either NTCP or TCP) calculated for an individual user-defined set of parameters and associated uncertainties.

### Analysis 2

The second analysis involved using the Marzi parameters and uncertainties for calculating the NTCP for late rectal toxicity with the aim of determining the impact of such clinically acceptable values in the final probability calculated. For this analysis, all the reported uncertainties were simultaneously considered.

## Results

**Figure 1:**
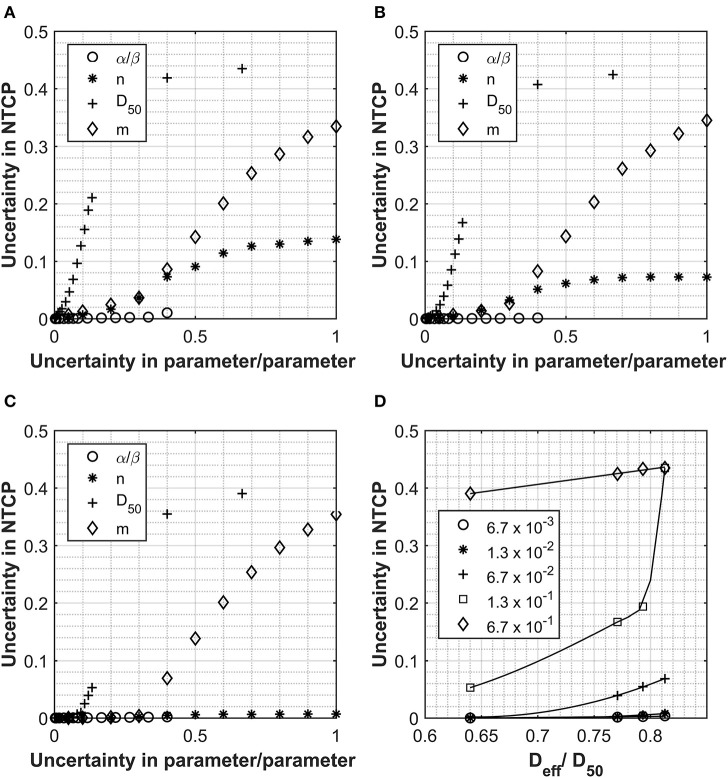
Data collected for Analysis 1, normal tissue complication probability (NTCP) calculations using the Lyman Kutcher Burman (LKB) model for the rectum. **(A–C)** The impact on the overall NTCP uncertainties as a result of increasing uncertainty in the individual parameters. Values used for α/β, n, D_50_, and m were 300 cGy, 0.1, 7,500 cGy, and 0.1, respectively, and the uncertainties applied are expressed as a fraction of each parameter. **(D)** shows the relationship between D_eff_ (as a fraction of the D_50_) and the uncertainty in the final NTCP calculated for different levels of uncertainty in the D_50_ parameter; lines are for guiding the eye only.

Figures 1A–C report how the uncertainties in the calculated NTCP values varies as a function of the uncertainties of the input parameters for three clinically approved treatment cases with different D_eff_ doses. Figure 1D shows how the D_eff_ as a percentage of the D_50_ parameter varies with uncertainty in NTCP for the four patient cases investigated. The different curves represent different levels of uncertainty applied to the D_50_-simulated parameter sets. For all cases, the uncertainty in the NTCP calculation is dominated by the uncertainty in the D_50_ parameter, and it follows a similar trend with its value initially increasing exponentially to then reaching a plateau (NTCP uncertainty ~42%) for D_50_ uncertainties >40%. There is an almost immediate increase in NTCP uncertainty for the patients with the higher D_eff_, while a slight lag is observed for the lowest D_eff_ patient. A similar trend is observed for the NTCP uncertainty as a function of the m parameter, although the impact is much smaller than for the D_50_ parameter. Interestingly, the other parameters (including the α/β ratio) play a much smaller role contributing at most 15% of the NTCP uncertainty for 100% uncertainty in the input parameter. The uncertainty in the α/β ratio had the least impact on the overall uncertainty in NTCP. Only the patient with the very highest D_eff_ showed a significant increase in NTCP uncertainty but that was small and not observed until the uncertainty in α/β reached 40%.

Considering the critical effect of D_eff_, the uncertainties in the NTCP have been reported as a function of the D_eff_/D_50_ ratio for different D_50_ uncertainties in Figure 1D. The data highlight how, for low D_eff_ plans, the uncertainty in NTCP are quite small irrelevant of the uncertainty in all the input parameters. However, as D_eff_ approaches 80% of the D_50_ value, the inaccuracy in determining the D_50_ has a major impact on the NTCP uncertainty. From Figure 1, it also emerges that an uncertainty of D_50_ < 6.7% would be required to maintain the NTCP uncertainty <5% irrespective of the D_eff_ values. The only exception is patient A (uncertainty in NTCP of 6.9%); however, this patient is at the upper limit of what would be accepted clinically for rectal doses.

**Figure 2:**
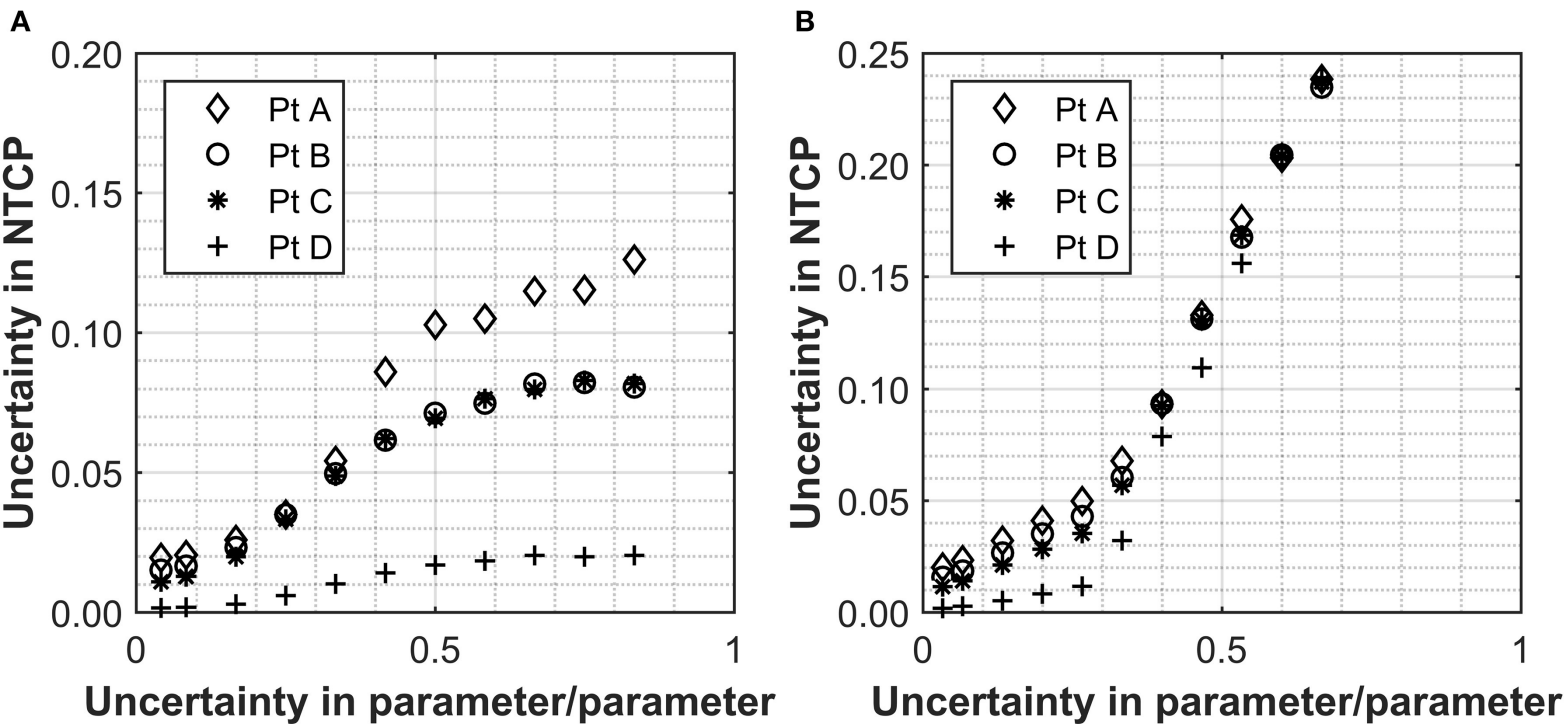
The relationship between the uncertainty in n **(A)** and m **(B)** with the overall uncertainty in the NTCP calculated for a selection of patients with ranging values of rectum Deff. Values used for α/β, n, D_50_, and m were 230 ± 60 cGy, 0.12, 7,600 ± 190 cGy, and 0.15, respectively (18). The uncertainties applied are expressed as a fraction in question and uncertainties are applied to either n or m individually with an uncertainty of zero used for the parameter not being assessed.

In order to appreciate the level of uncertainties, which commonly affect clinically relevant NTCP estimations, the data set from Marzi et al. was used on the four patient cases highlighted above. This data set was selected as it investigated NTCP for a relevant biological endpoint and was one of the fewer studies quoting uncertainties on input parameters, 2.5 and 26% for D_50_ and α/β, respectively. Without further uncertainty considerations for the other parameters, the overall NTCP uncertainties calculated through the simulation approach are of the order of 2% for all cases investigated (Figure 2). This is significantly lower than the uncertainty that would result from a simple relative error propagation (i.e., square root of the sum of the individual relative errors squared), which would be dominated by the error in the α/β resulting in the overall uncertainty for the NTCP values of ~26%. Moreover, the impact of uncertainties in the n or m parameters (the former in particular) become quickly significant pushing the NTCP uncertainty up to ~10% for an input parameter error of 40% [Figures 2A (n), B (m)]. The effect was again more pronounced for patients with high D_eff_. For small uncertainties in m or n, the uncertainty in NTCP derives mainly from the D_50_ uncertainty, and the differences in the uncertainties between the patients are a consequence of the different D_eff_ values.

As expected, the uncertainties in the input parameters combine for the overall NTCP calculations. For the patient with the highest D_eff_, a D_50_ uncertainty alone of 2.5% (as for the Marzi data set) would result in an NTCP uncertainty of 1.9%, which remains the same when combined with the uncertainty in α/β (up to ~26%). With a 26% uncertainty in the α/β alone, the uncertainty in the final NTCP parameter is 0.3% confirming that D_50_ is the dominant source of uncertainty where the fractionation regime is at, or close to, 2 Gy/fraction. This clearly shows that impact of the individual parameters is not linear with regard to uncertainty propagation.

**Figure 3:**
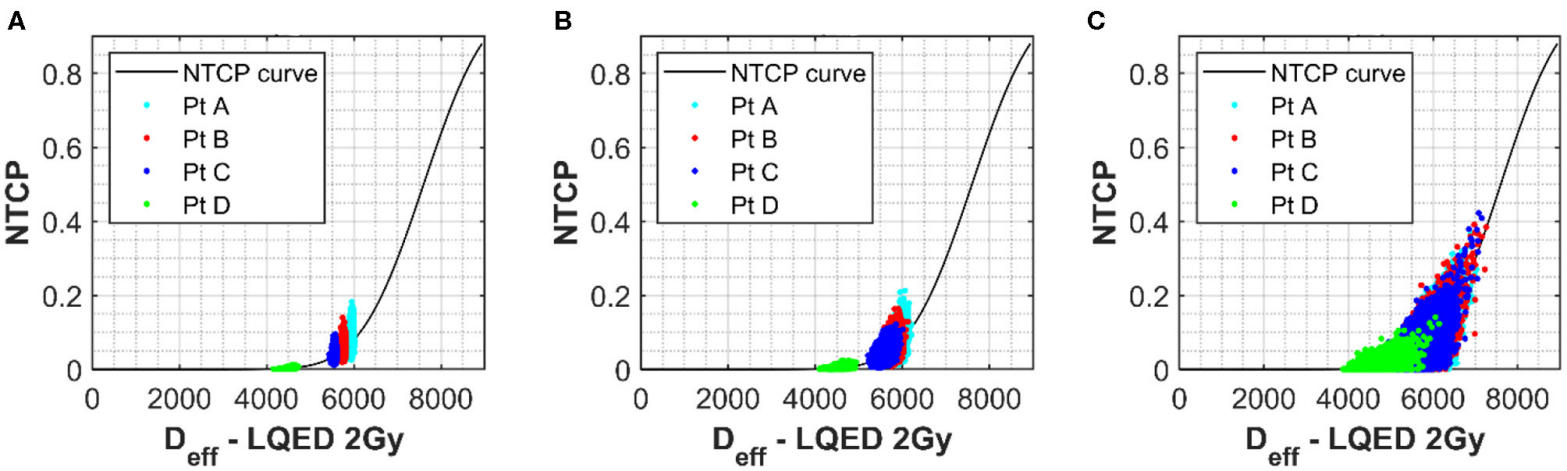
**(A)** The NTCP vs. D_eff_ curve for grade 2 late toxicity of the rectum (18). Values used for α/β, n, D_50_, and m were 230 cGy, 0.12, 7,600 cGy, and 0.15, respectively. Simulated rectum NTCP values for a selection of patients with uncertainties of 60 and 190 cGy applied to α/β and D_50_ parameters, respectively, have been plotted onto the curve for a selection of patients with different D_eff_. Each point represents one simulation. **(B,C)** The simulated results when an additional uncertainty is applied to both m and n of 0.1 **(B)** and 0.3 **(C)**.

In order to better appreciate the interlink between the input parameter uncertainties, variation in the NTCP values have been simulated for the four different clinical cases assuming uncertainties on all the parameters simultaneously. The Marzi data set was again used as starting point, and errors of ~7 or ~20% were added to both the n and m parameters. Data in Figure 3A (no uncertainty on m or n) clearly show the impact of uncertainty in the D_50_ and α/β on the NTCP values with minimum effect on the D_eff_. It is also interesting to notice how the NTCP values are not symmetrically distributed around the NTCP curve but stretched toward the high NTCP values. This would have strong consequences for the setting of NTCP acceptance levels for a population case. The addition of uncertainties in the n and m parameters (see Figures 3B,C) increases the D_eff_ values moving the calculations toward the steeper part of the NTCP curve resulting, therefore, in higher NTCP values. While an individual ~20% uncertainty in the n or m parameters had only a small effect on the NTCP uncertainty when D_50_ ~2.5% (Figures 3A,B), their combined effect pushes the NTCP values from <15% up to 40% despite a still low D_50_ uncertainty (Figure 3C). Therefore, when considering the combined uncertainties, it is important to keep the uncertainty in the m and n parameters below 0.3 for both parameters.

For a greater understanding of the overall picture, the range in the NTCP values calculated was also evaluated. The NTCP range without uncertainties applied to the m and n parameter was 0.03–0.21. This range increases to 0.01–0.21 for an uncertainty of 0.1 in m and n and then to 0–0.37 for an uncertainty of 0.3. This demonstrates a clear benefit in keeping the uncertainty for such parameters in the lower range (around the 0.1 mark) where the influence of these parameters is low.

Interestingly, a conventional error propagation approach would result in an average NTCP uncertainty of ~28% for the input parameter set: D_50_ = 7,600 ± 190, α/β = 230 ± 60, n = 0.12 ± 0.01 and m = 0.15 ± 0.01, while the simulation approach estimates an uncertainty of ~40% for the selected patients. The average NTCP values also change when accounting for uncertainties using the simulation approach due to the low-level boundary of NTCP = 0 and the data spread, which pushes the NTCP values up. Table 1 shows the difference in the NTCP values and their related uncertainties comparing a conventional error propagation method to the simulation approach.

**Table 1:** Table showing the differences in both mean and uncertainty (standard deviation expressed as a percentage of the mean) for different methods of error propagation calculation, the conventional numerical method, and the simulated method discussed in this manuscript.

	**Conventional error propagation**		**Simulation approach**	
	**NTCP**	**Error [%]**	**NTCP**	**Error [%]**
Patient A	0.0733	28	0.0761	32
Patient B	0.0535	28	0.0557	36
Patient C	0.0367	28	0.0391	41
Patient D	0.0040	28	0.0046	63

**Figure 4:**
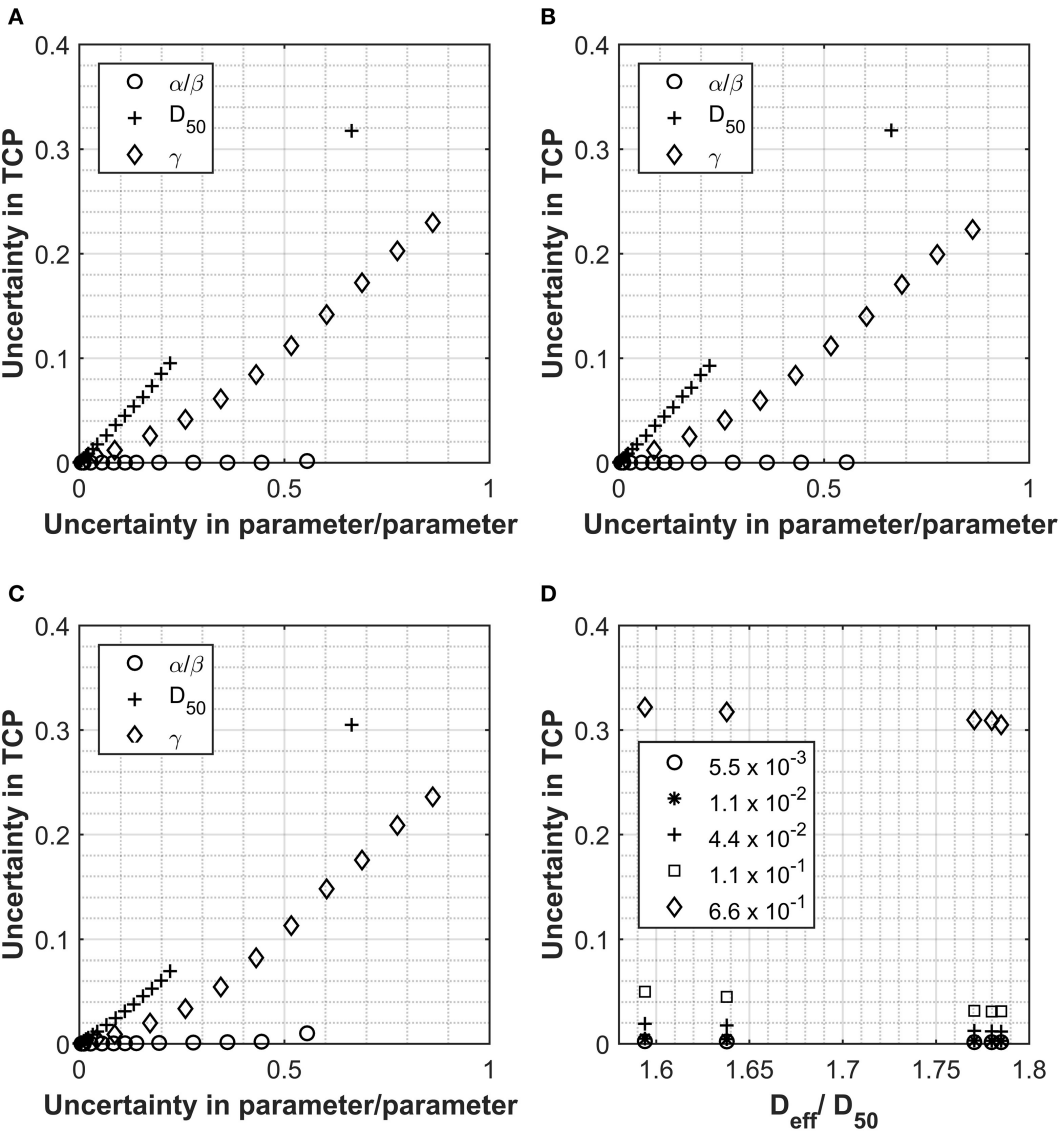
Data collected for Analysis 1, tumor control probability (TCP) calculations using the Lind model for the prostate and prostate PTV. PTV and prostate data displayed in Panel **(A, B)** respectively for Pt A. Patient B PTV data displayed in Panel **(C)**. Panels show the impact on the overall TCP value as a result of increasing individual parameters. Values used for α/β, D_50_ and γ were 180 cGy, 4518 cGy and 1.16 respectively and the uncertainties applied are expressed as a fraction of each parameter. Panel **(D)** shows the relationship between D_*eff*_ (as a fraction of the D_50_) and the uncertainty in the final TCP calculated for different levels of uncertainty in the D_50_ parameter.

A similar approach has been also used to investigate the impact of the input parameter uncertainties on the TCP calculations (see Figures 4A–D). Each input parameter was individually considered for the four cases used so far and the relationship between uncertainty in TCP plotted against the uncertainties applied to the individual input parameters. The PTV was used as opposed to the prostate volume due the availability of data. For the one patient data set where both PTV and prostate volume were present, an analysis was performed with both structures, and data were very similar (see Figures 4A,B). D_50_ again appeared to be the most critical parameter, however, following a more linear response than for the NTCP investigations. The relationship between TCP and uncertainty in parameter appeared to be almost linear for all parameters and almost identical for all D_eff_ analyzed. The relationship D_eff_/D_50_ vs. TCP uncertainty showed a slight benefit for the higher dose structures (see Figure 4D). From the limited range of cases investigated, uncertainty levels <10% for D_50_ would be required to achieve TCP uncertainties <5%. This could also be of importance when considering co-factors, e.g., clinical factors such as age, concurrent chemo, prior surgery, etc. A study on the impact of co-factors (20) dealt with differences in response through the D_50_ parameter. Using an uncertainty analysis such as described could be used clinically to define a level (uncertainty in D_50_) at which it is appropriate to separate sub-groups out of a main group.

Uncertainties in the α/β parameter appear to have a negligible impact on the TCP uncertainty for the Lind model. This is due to the fractionation regime used and the fact that the influence of the α/β ratio is in converting from physical dose into LQED2. Owing to the fact that most of the voxels in the PTV will be receiving a fractionation of very close to 2 Gy per fraction, physical dose is almost identical to LQED2, which means that the parameter has very little impact on the structure dose being evaluated with this model. The only response observed were for patients B and C (Figure 4C), where there was a small impact on TCP uncertainty after an uncertainty in the parameter of greater than 0.5. These patients had the highest Deff and, therefore, the highest dose per fraction (~2.2 Gy/fraction), which is the farthest dose/fraction from 2 Gy, which is the fractionation that LQED2 is referenced to. While the behavior observed with these test cases would hold true for a large majority of treatments, it would not be the case for all. With the move toward more biologically driven treatments [e.g., use of biological treatment volume (BTV) and also dose escalation to parts of the tumor] (21), far less homogenous treatments are used with large variations in dose across the PTV; these could exceed 130%. In such cases, not all voxels within the PTV will have doses at, or around, 2 Gy/#, and this will influence the impact of alpha-beta ratio uncertainty.

**Figure 5:**
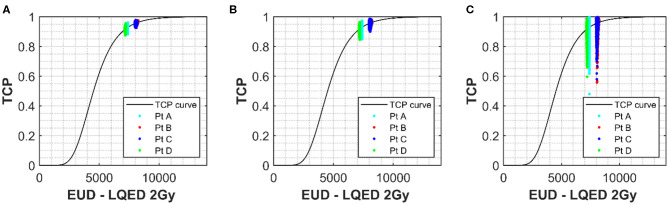
The solid line shows the TCP vs. D_eff_ curve for the prostate PTV using values of 180 cGy, 4,518 cGy, and 1.16 for α/β, D_50_, and γ respectively (18). Simulated prostate TCP values for a selection of patients with uncertainties of 47 cGy (26%) and 113 cGy (2.5%) applied to α/β and D_50_ parameters, respectively, have been plotted onto the curve for a selection of patients with different D_eff_. **(A)** Shows no uncertainty applied to m for the simulated patients and **(B,C)** show uncertainties in γ of 0.1 and 0.3 respectively. D_eff_ for each simulation was calculated using the generalized equivalent uniform dose (EUD) formula, with parameter a set to –10.

Figures 5A–C show the shape of the TCP curve using prostate tumor parameters from Okunieff et al. (19). Similar to the NTCP analysis, uncertainties of 2.5 and 26% were applied to D_50_ and α/β, respectively, and the simulated data were plotted on the graph. Uncertainties of 0, 0.1, and 0.3 were applied to the γ parameter and are shown in Figures 5A–C, respectively. A value of −10 was selected for the “a” parameter to convert the DVH dose into an equivalent uniform dose (EUD), which is identical to D_eff_, with n = 1/a. This value is widely used for tumor structures, and due to the fact that the variation in the dose distribution (from the DVH) for the structure is low (1% variation), it was thought to be an appropriate choice (22). Of course, in cases where there is dose escalation in the PTV, an appropriate value for the parameter “a” would have to be considered more carefully. There is little spread in the x-direction axis due to the fact that the only parameter with influence on dose is the α/β. As mentioned earlier, where the fractionation regime is close to a standard one delivering 2 Gy/fraction, the α/β has little impact on the resulting LQED2 Gy calculated. Patients B and C have the largest spread because their dose/fraction is farthest away from the standard 2 Gy/fraction. However, while their x-axis uncertainty is larger, the overall uncertainty in the TCP is lower (e.g. 3.5% for Patient C (less standard dose/fraction) compared with 4.4% for Patient D (standard dose/fraction) for an uncertainty in γ of 30%), which is in keeping with the relationship shown in Figure 4D, where the higher-dose PTVs benefit from slightly improved uncertainties in TCP for different levels of uncertainty in the D_50_ parameter. As for the NTCP data, the combined effect of uncertainties in the input parameters quickly results in a significant increase in the TCP range. The TCP value range using an uncertainty in γ of 30% is high (~48 to 99%) compared to a range of 88 to 96% for 0 uncertainty in γ, which increases to 85 to 97% for uncertainty in γ of 10%. While the actual TCP uncertainty (calculated as standard deviation of the probability data collected) is under 5% for all patients, the spreading of the range data shows a clear benefit (similar to the LKB for the NTCP data) of keeping uncertainties in the γ below 10%.

There was a dose effect with structures having D_eff_ or EUD at the periphery of the slope, having smaller uncertainty than those nearer the center of the curve. This is due to the fact that positions on the curve that are at the start or end of the curve are on a shallower gradient and, therefore, less impacted by changes in the slope. This software can be used as a tool to highlight where models maybe susceptible to steep increases in uncertainty size, e.g., for LKB, there are certain boundaries around which you may need to be especially careful; however, for the Lind model, there seems to be an almost linear increase in uncertainty in TCP for increasing uncertainty of the input parameters. The analysis also indicates the desired level of uncertainty for the input biological parameters in order to obtain TCP/NTCP values with reasonable confidence intervals. Such information can be used to focus future research efforts and improve estimation of biological parameters, which play a key role in TCP/NTCP models.

## Conclusion

We have developed software, which allows an estimation of the uncertainty associated with TCP/NTCP predictions. The approach can provide insight on the uncertainty associated with TCP and NTCP calculations as a function of the uncertainty for the biological input parameters, the patient specific anatomy and the treatment dose. The software has been used to identify the dominant parameters (D_50_ for both models tested) with respect to uncertainty propagation. A conventional basic error propagation approach was also carried out, and it appeared to underestimate the error in the final NTCP/TCP values suggesting that different approaches should be considered. Owing to the fractionation regime of the treatment plans used for the study, there was little impact from the α/β parameter. For future work, there is a need to evaluate the impact for cases where the fractionation regime is significantly different, e.g., CHHIP trial and also in cases where inhomogeneous dose distributions are delivered to the PTV. This could soon be the norm especially now that imaging modalities are in place to identify such areas within the PTV with, e.g., increased radiosensitivity, increased clonogen density, and areas of increased hypoxia that would benefit from escalated doses. As we are moving into an era of highly conformal treatment planning, dose escalation, and novel approaches, such as dose painting, including radiobiological guidance as part of the optimization process, has been proposed to help inform the evaluation of the trade-off between tumor control and normal tissue toxicity (21, 23–25). Including uncertainty will allow evaluation and optimization of the robustness of plans to biological variations. Similarly, the algorithm can be used as a useful tool to compare radiobiological models both in terms of sensitivity and, through application to clinical studies, accuracy and guide further developments. The present approach estimates the errors on the NTCP/TCP values by simulating a random population of input parameters uncorrelated but each with constrains of their individual uncertainties. Future work will look at the inter-dependency of input parameter errors using Bayesian approaches. An additional useful feature to include in this software would be to incorporate an uncertainty in the dose itself. Currently, the software only looks at the probability in input parameters; however, there is an uncertainty on dose from many contributing factors. It would be useful to be able to characterize the uncertainty in the DVH and incorporate dose uncertainties in the TCP/NTCP uncertainties. Finally, the study will also be extended to allow analysis of data from other TCP and NTCP calculations that incorporate the α/β directly into the models to a greater extent and not just through converting physical dose to LQED2, e.g., the Webb model (22).

## Data Availability Statement

The datasets generated for this study are available on request to the corresponding author.

## Author Contributions

GS, MB, and MH contributed to the conception and design of the study. MatLab software was written by MB. Implementation of the model was checked by MH and GS. Eclipse and Raystation plans and DVHs were prepared by MH. Simulations and data preparation for the manuscript were carried out by MB. Content discussion, writing/revision of the manuscript was carried out by MB, GS, and MH. All authors contributed to the article and approved the submitted version.

## Conflict of Interest

The authors declare that the research was conducted in the absence of any commercial or financial relationships that could be construed as a potential conflict of interest.
