# Corrigendum: Evaluating the Propagation of Uncertainties in Biologically Based Treatment Planning Parameters

**DOI:** 10.3389/fonc.2020.603976

**Published:** 2021-01-27

**Authors:** Miriam A. Barry, Mohammad Hussein, Giuseppe Schettino

**Affiliations:** ^1^ National Physical Laboratory, Medical Radiation Science, Teddington, United Kingdom; ^2^ Department of Physics, University of Surrey, Guildford, United Kingdom

**Keywords:** normal tissue complication probability (NTCP), tumor control probability (TCP), uncertainty, biologically based treatment planning, biological optimization

The following corrections have been made to the original article.

Correction in Equation 1

In the original article, the first term of the Equation 1, D_i_, was omitted**.** The corrected Equation 1 appears below.


LQED2i=Di·1+Dinαβ1+2αβ

Text Corrections

In the original article, the following typographical errors have been corrected.

Instances where tumor control probability (TCP) has been incorrectly written as tissue control probability, and normal tissue complication probability (NTCP) has been incorrectly written as normal tissue control probability. These instances were in the Abstract and Figure 1 caption.

In **Results Paragraph 2**, the following corrections have been made: Patient A was incorrectly referred to as Patient 4.

In **Results Paragraph 8**, the sentence: “A similar approach has been also used to investigate the impact of the input parameter uncertainties on the TCP calculations (see **Figures 4A, D**)” it should read (see **Figures 4A-D**). The sentence “The relationship D_50_ vs. TCP uncertainty showed a slight benefit for the higher dose structures (see **Figure 4D**).” should read “The relationship D_eff_/D_50_ vs. TCP uncertainty showed a slight benefit for the higher dose structures (see **Figure 4D**).”

**Figure 4:**
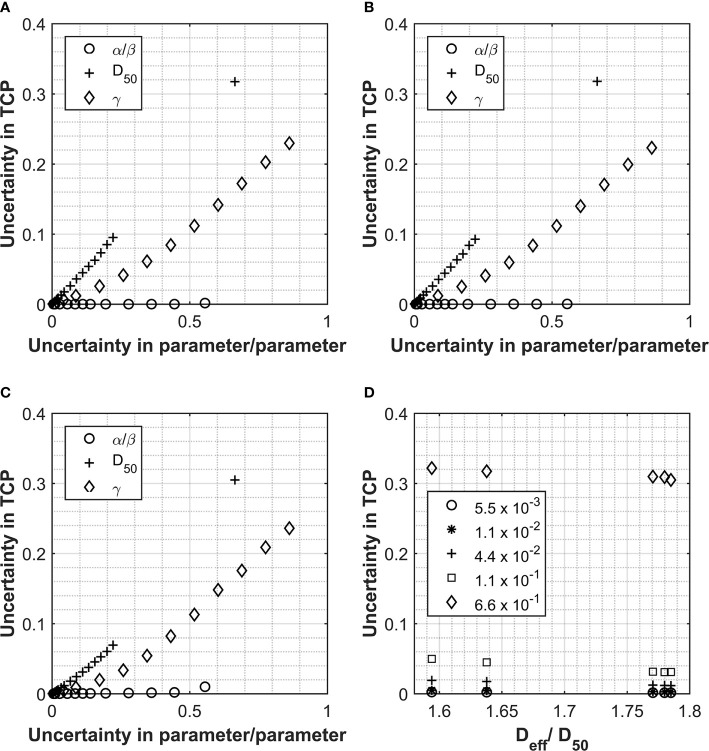
Data collected for Analysis 1, tumor control probability (TCP) calculations using the Lind model for the prostate and prostate PTV. PTV and prostate data displayed in Panel **(A, B)** respectively for Pt A. Patient B PTV data displayed in Panel **(C)**. Panels show the impact on the overall TCP value as a result of increasing individual parameters. Values used for α/β, D_50_ and γ were 180 cGy, 4518 cGy and 1.16 respectively and the uncertainties applied are expressed as a fraction of each parameter. Panel **(D)** shows the relationship between D_eff_ (as a fraction of the D_50_) and the uncertainty in the final TCP calculated for different levels of uncertainty in the D_50_ parameter.

In **Results Paragraph 9**, The word **NTCP** in sentence: “The only response observed were for patients B and C, where there was a small impact on NTCP uncertainty after an uncertainty in the parameter of greater than 0.5.” should have been **TCP**. The cross reference to **Figure 4C** has been added after “..patients B..”.

In **Results Paragraph 10**, the slope parameter was referred to as m instead of γ in the sentence: “Uncertainties of 0, 0.1, and 0.3 were applied to the m parameter and are shown in Figures 5A–C, respectively”. The sentence “Patients C and D have the largest spread because their dose/fraction is farthest away from the standard 2 Gy/fraction” should read “Patients B and C have the largest spread because their dose/fraction is farthest away from the standard 2 Gy/fraction”. Additional text (for clarification) is added to this sentence “However, while their x-axis uncertainty is larger, the overall uncertainty in the TCP is lower (3.5% for Patient C compared with 4.4% for Patient D for an uncertainty in γ of 30%),…” to make it read “However, while their x-axis uncertainty is larger, the overall uncertainty in the TCP is lower (e.g. 3.5% for Patient C (less standard dose/fraction) compared with 4.4% for Patient D (standard dose/fraction) for an uncertainty in γ of 30%),.”.

In **Conclusion Paragraph 1** The word TPC in the sentence “The present approach estimates the errors on the NTCP/TPC values.”, should be TCP.

Correction to Figure 4

There was a mistake in **Figure 4** as published. Panel B displayed the graph for the PTV not the prostate and an error was found in the D_eff_/D_50_ value calculated for Pt B. There was a mistake in the legend for **Figure 4** as published. The slope parameter is referred to as m instead of γ. There was a mistake in the caption for **Figure 4**. The first sentence was modified to address errors in referencing to individual panels. The word tissue should be tumor, TC should be TCP and the parameter m should have been referred to as parameter γ. A final sentence has been added to describe panel D. The corrected **Figure 4** (including corrected legend) and caption appear below.

Correction to Figure 5 Caption

There was a mistake in the caption for **Figure 5** as published. The parameter γ was referred to as m throughout the caption, the parameter α/β was referred to as a/b. Also “prostate” should read “prostate PTV”.

The authors apologize for these errors and state that these do not change the scientific conclusions of the article in any way. The original article has been updated.

