# Supplementary material for: Evaluating the Propagation of Uncertainties in Biologically Based Treatment Planning Parameters
Source: Front Oncol. 2020 Jul 21;10:1058. doi: 10.3389/fonc.2020.01058 (PMC7386327; doi:10.3389/fonc.2020.01058)
Supplement: Supplementary file 1 [file Data_Sheet_1.docx]

Supplementary Material

# MatLab™ Software Description

The functionality of the MatLab™ software is described below and key steps displayed in the figures. Initially, the DVH format is selected, see Supplementary Figure1. Currently the DVH formats that can be used are BioSuite^©^, Eclipse^TM^, Pinnacle^TM^ and RayStation^TM^. Once the format has been selected, the user can click on the “Upload DVH file” button which allows browsing for the DVH required. Once the file has been selected, a drop-down menu populates which allows to the selection of the organ of interest, see SupplementaryFigure2. Once the organ has been selected, the fields required to convert from physical dose into LQED 2 Gy will be available, see SupplementaryFigure3. By separating with a space, it is possible to input both an alpha-beta ratio parameter (first value) and an associated uncertainty (second value) if required. If an uncertainty is applied to the alpha-beta ratio then the normally distributed random number generator in MatLab is used to create a 1 x n vector of alpha-beta values (where n is a user defined value entered into the “Number of iterations” field) that has a mean value and standard deviation over the entire vector matching the mean and standard deviation entered. The “Number of iterations” determine the number of values simulated by the MatLab random number generator. The “Prepare DVH” data, see SupplementaryFigure4, converts the physical DVH to a single DVH in terms of LQED2 (for a single given value of alpha beta) or multiple DVHs, one for each value of alpha-beta in the 1 x n vector containing the simulated sample of alpha-beta values. Once all the physical and LQED2Gy DVH data has been either loaded or calculated, a biological model can be selected from the drop-down menu as shown in SupplementaryFigure5. Currently the models available are LKB (NTCP), Lind (TCP) and Webb (TCP) although more can be added. Once the required model has been selected, the required parameter fields appear. It is possible to account for an uncertainty for a particular parameter by first entering the parameter value followed by (in the same field) the uncertainty, separating the two values with a space as before when applying an uncertainty to the alpha-beta parameter, see SupplementaryFigure6. Finally, the “Calculate” button compute the probability result, see SupplementaryFigure7. If uncertainty on any one of the parameters has been entered (either for in the calculation of the LQED2 or for the probability model itself) a vector (1 x n) of probability results will be produced from which the mean and standard deviation (over the vector) is calculated and displayed.

Additional functionality include a save button which allows data from the results vector to be saved to an excel file alongside all user inputs for that calculation. Parameters can also be cleared at the different stages to reduce the number of times data has to be re-loaded into the program. There is a lower stage where just the model type and associated parameters can be cleared to allow different models or different parameters to be analyzed for a loaded set of physical and LQED2Gy corrected DVH(s). There is also a higher level clear where everything is cleared enabling a completely new simulation to be run from the initial step of importing a DVH.


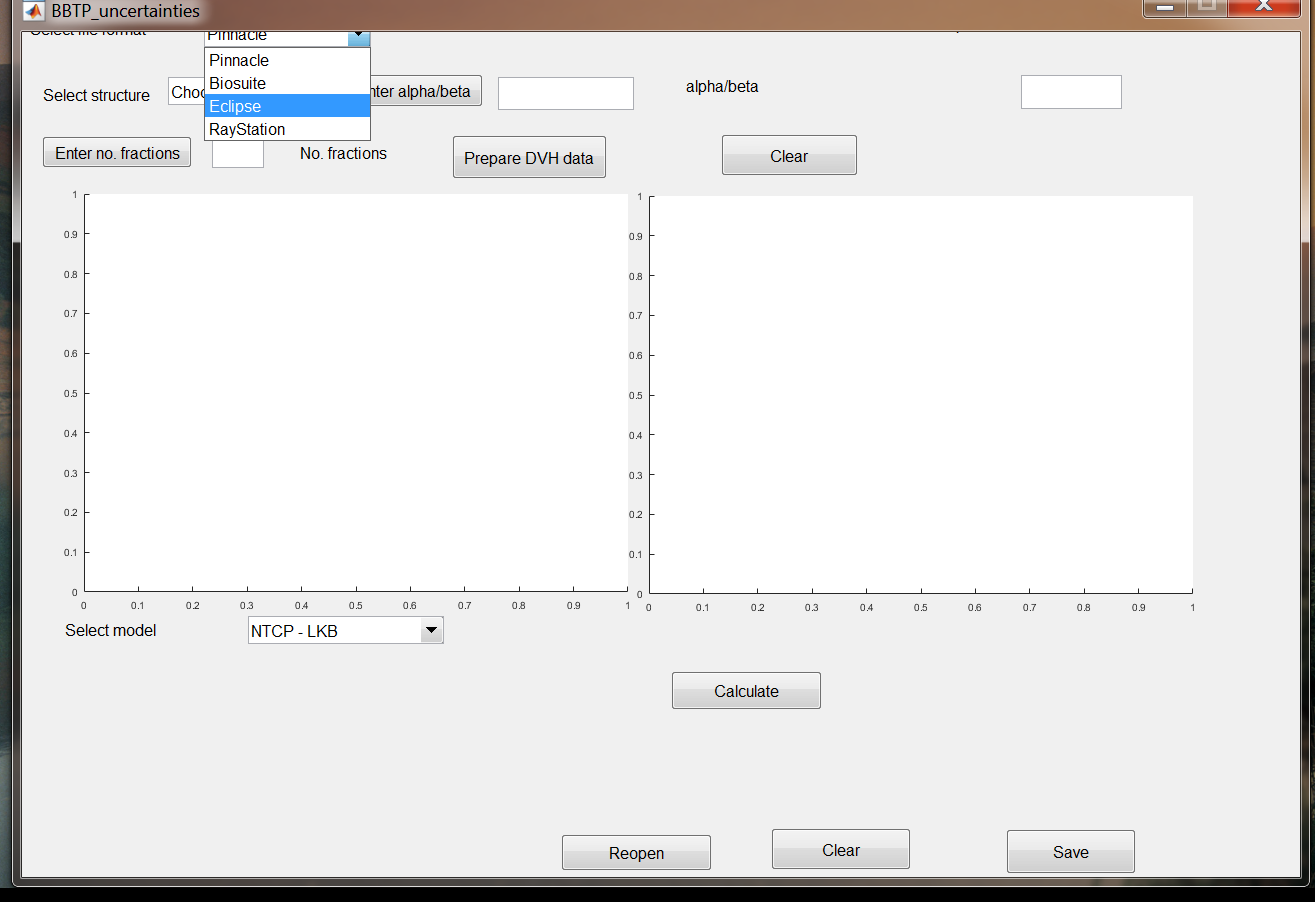


**Supplementary Figure 1.** Initially the DVH format is selected.


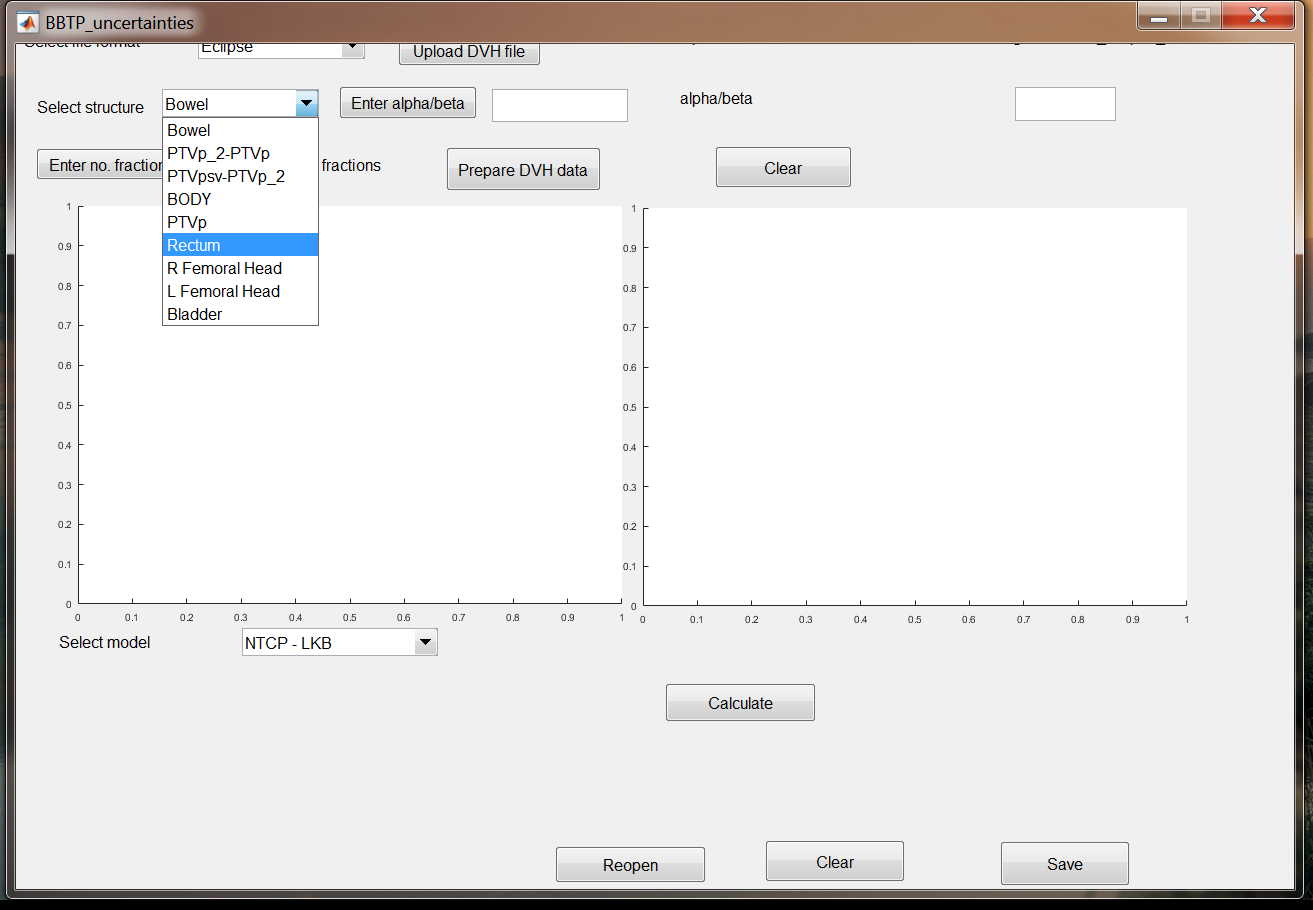


**Supplementary Figure 2.** Once the DVH has been imported a drop-down menu automatically populates allowing you to select the organ of interest.


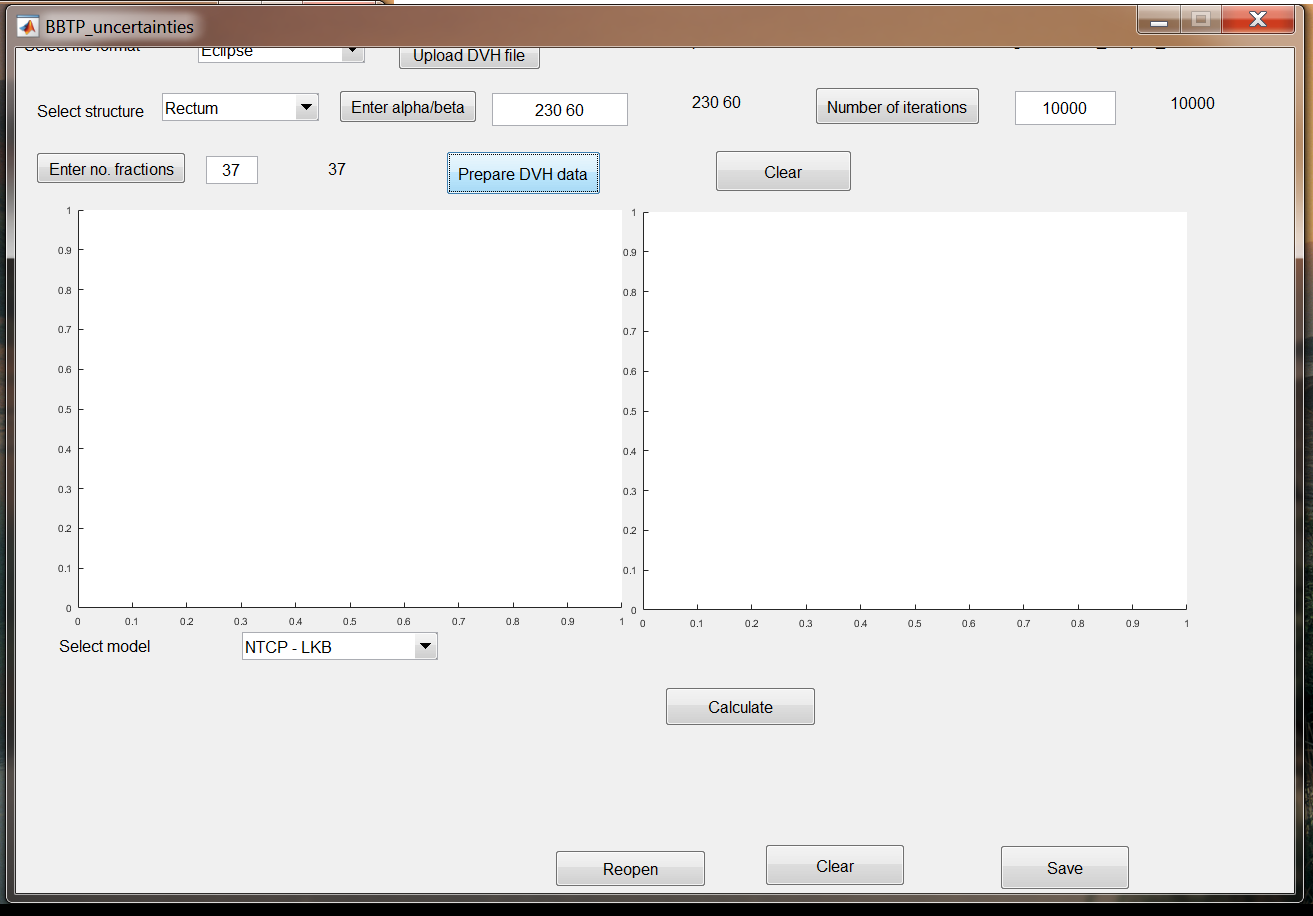


**Supplementary Figure3.** Enter visible fields with required information to enable the LQED 2Gy to be calculated for the DVH loaded. If an uncertainty is to be evaluated in any parameter, the “Number of iterations” field must be populated. This will define the number of values to be simulated for the parameter(s) being evaluated.


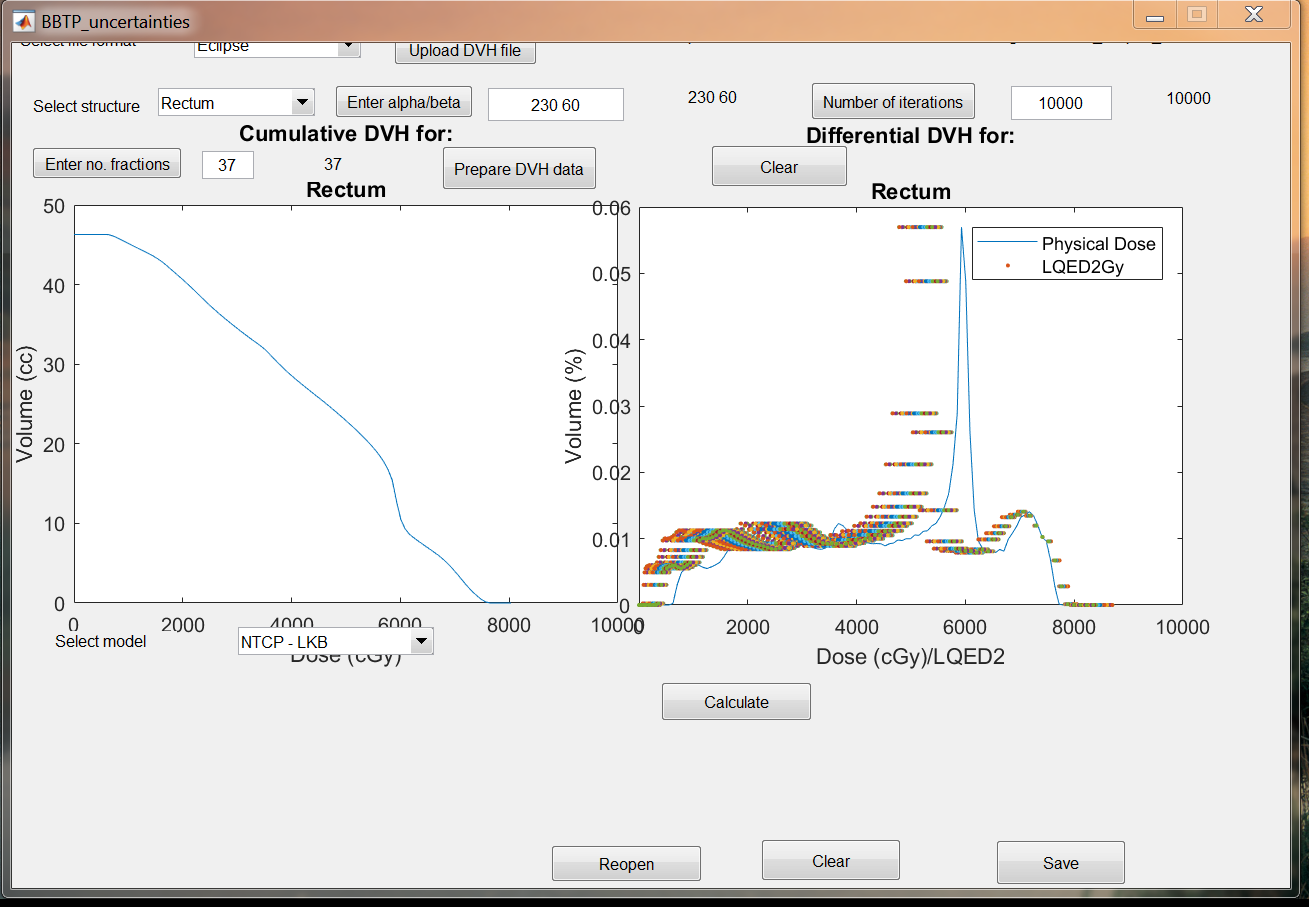


**Supplementary Figure4.** Pressing the “Prepare DVH data” will display the physical DVH and also convert to LQED2 based on the alpha-beta ratio value or range of values.


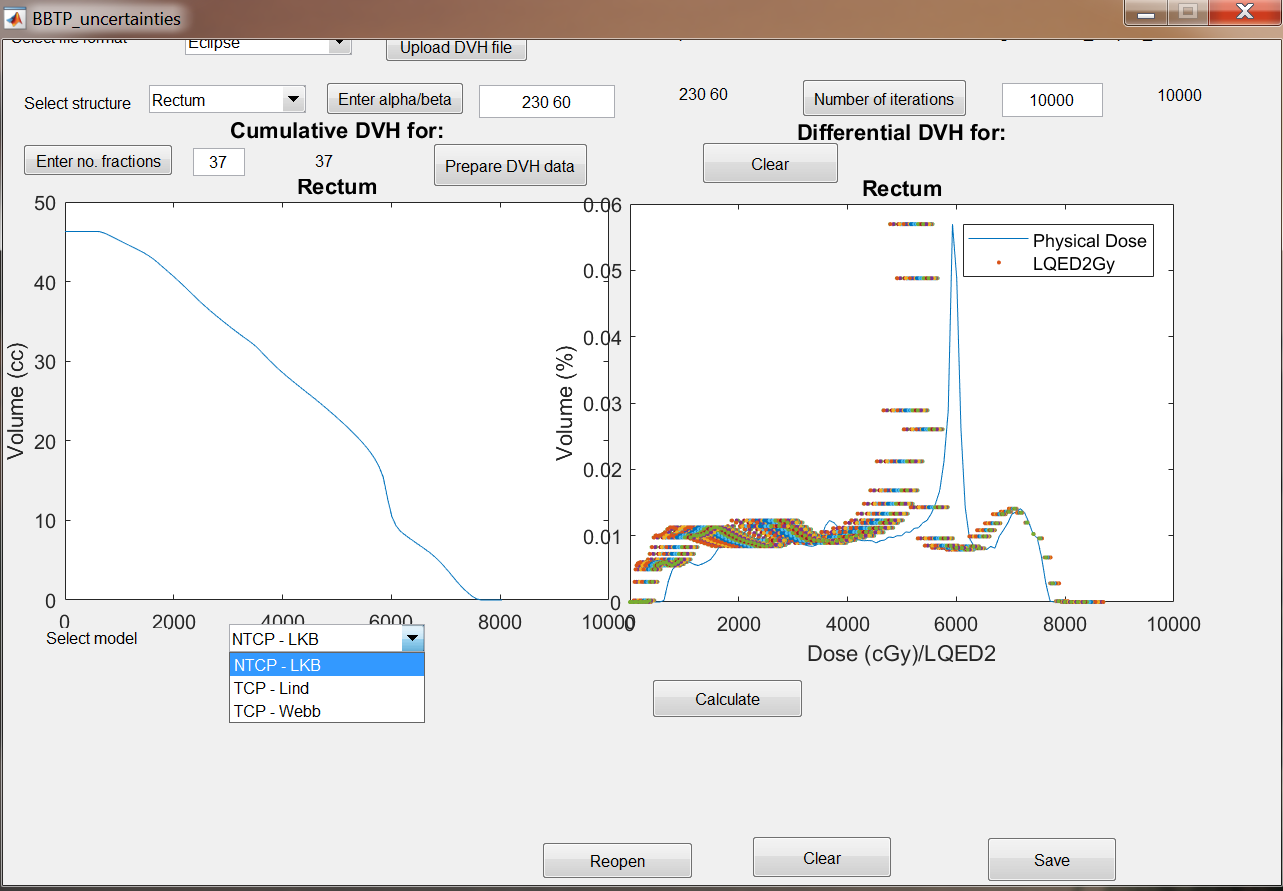


**Supplementary Figure5.** Once the physical and LQED2 doses are loaded/calculated, a TCP or NTCP model can be selected from the drop-down box.


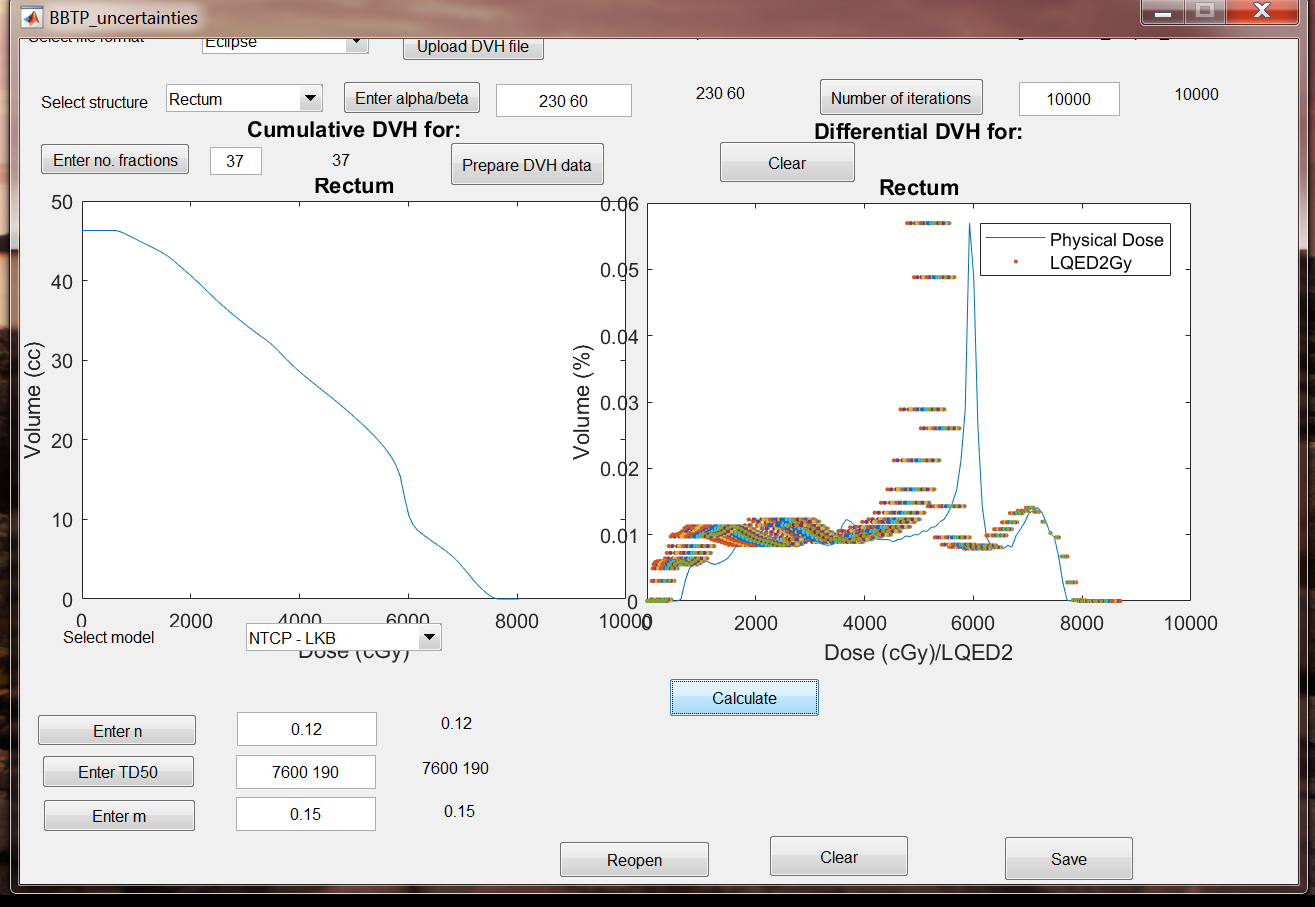


**Supplementary Figure6.** Once the model has been selected, the required parameters can be inserted in the fields that appear. An uncertainty in any parameter can be included in the field, after the parameter value, separating the two values with a space.


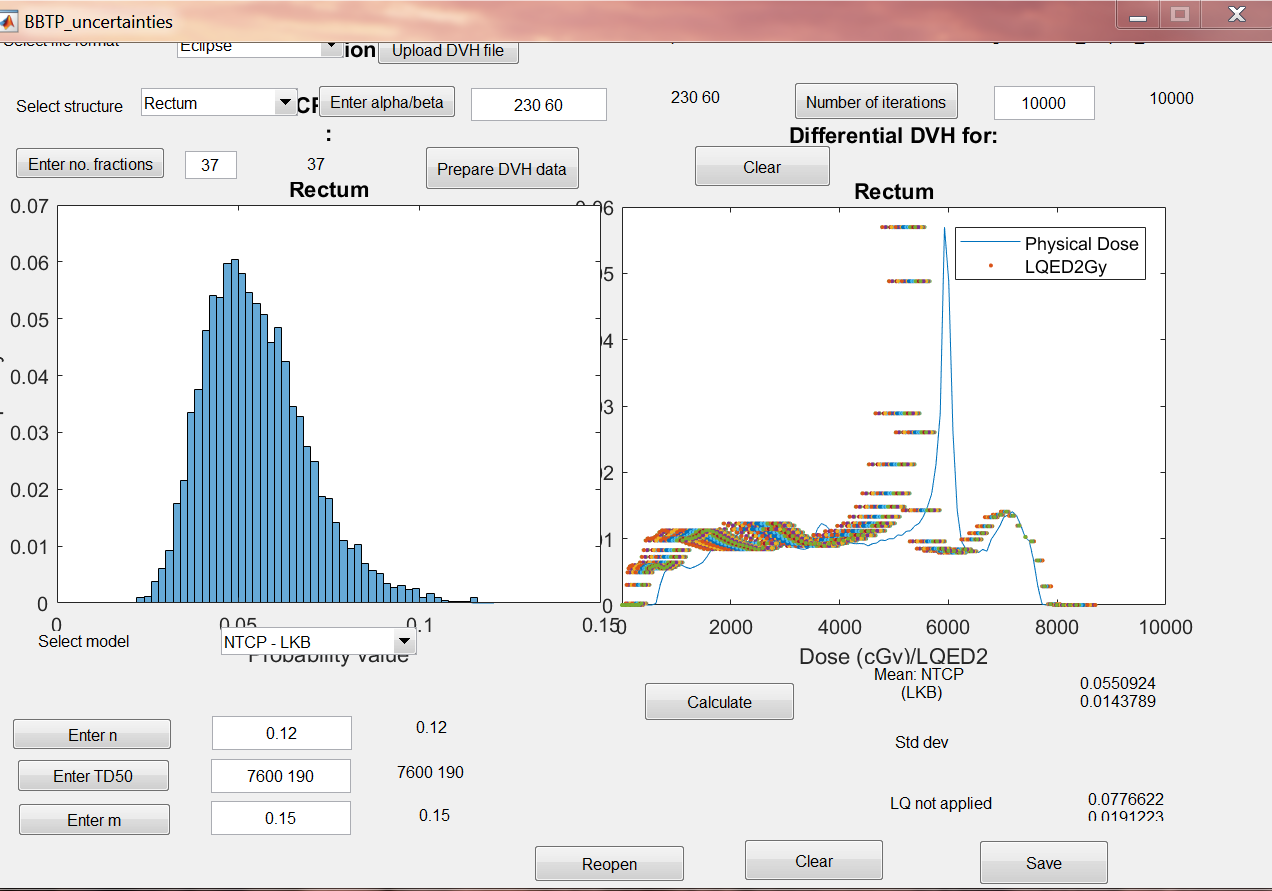


**Supplementary Figure7.** Pressing the “Calculate” button will calculate the probability from the DVH data and the parameters entered. If an uncertainty has been applied to any parameter, a standard deviation will be displayed alongside the mean TCP or NTCP value. The upper value(s) are related to the LQED2 corrected doses and the value(s) below relate to the physical dose.
